# C-751-01. A Decision Aid for Burn Survivors Considering Reconstructive Scar Surgery

**DOI:** 10.1093/jbcr/irag033.006

**Published:** 2026-04-06

**Authors:** Lea Elder, Rana Stephan, Molly Furcht, Clifford C Sheckter

**Affiliations:** Santa Clara Valley Healthcare System, San Jose, California; Santa Clara Valley Medical Center, San Jose, California; Santa Clara Valley Medical Center, San Jose, California; Stanford University, San Jose, California

## Abstract

**Introduction:**

Scar procedures including laser and surgery aim to improve quality of life in burn survivors, though some patients may have decisional regret or outcomes that do not meet expectations. Decision aids are tools used in pre-surgical counseling to improve patient decision making and decrease decisional regret. We aimed to create a decision aid for burn survivors regarding procedural scar treatment.

**Methods:**

We conducted semi-structured interviews with key stakeholders including reconstructive surgeons, psychologists, occupational/physical therapists, burn survivors, and family members of burn survivors. Using thematic analysis, we identified common themes related to decisional regret, expectations, and perceptions of success in reconstructive scar procedures. We developed a decision aid following the Ottawa Decision Support Framework using qualitative research methods. The decision aid was piloted with five patients undergoing reconstructive scar surgery.

**Results:**

40 participants were interviewed including 26 burn survivors and family members, 2 psychologists, 6 burn therapists, and 6 surgeons. Six themes were distilled from burn survivors/families: appreciating mixed expectations, understanding functional improvements are greater than cosmetic, investing in patient-physician relationship/trust, understanding procedural pain/recovery may be intense, preparedness for significant life interruption with temporary loss of independence, and need for adherence to postoperative plan. 79% of survivors felt surgery met or exceeded expectations while 21% felt regret about scar surgery. Five themes emerged from burn practitioners: setting realistic expectations, burn survivor engagement in surgical process/recovery, waiting for the proper timing to achieve best outcomes, prioritizing mental health before scar surgery, and investing in patient-physician relationship/trust. The decision aid has six sections: get the facts, compare options, your feelings (fig. 1), your decision, quiz yourself, and your summary. Of the five patients who piloted the decision aid, all proceeded with scar surgery and found the tool useful, reporting no regret at 6 weeks after surgery.

**Conclusions:**

A decision aid for scar surgery was successfully created using qualitative research methods. Burn survivor and burn practitioner domains reveal multiple opportunities for better expectation setting around surgery. Future research could employ this decision aid on a larger number of patients to determine clinical effectiveness in reducing regret and satisfaction with outcomes.

**Applicability of Research to Practice:**

Burn centers providing reconstructive scar procedures may consider incorporating this tool to augment surgical decision making and reduce the likelihood of regret and expectation mismatch.

**Funding for the study:**

Plastic Surgery Foundation.

